# The TRPA1 Ion Channel Contributes to Sensory-Guided Avoidance of Menthol in Mice

**DOI:** 10.1523/ENEURO.0304-19.2019

**Published:** 2019-10-30

**Authors:** Christian H. Lemon, Jordan E. Norris, Bradley A. Heldmann

**Affiliations:** Department of Biology, The University of Oklahoma, Norman, OK 73019

**Keywords:** ingestive behavior, menthol, trigeminal, TRP channels

## Abstract

The flavoring agent menthol elicits complex orosensory and behavioral effects including perceived cooling at low concentrations and irritation and ingestive avoidance at higher intensities. Oral menthol engages the cold-activated transient receptor potential (TRP) ion channel TRP melastatin 8 (TRPM8) on trigeminal fibers, although its aversive feature was discussed to involve activation of TRP ankyrin 1 (TRPA1) associated with nociceptive processing. Here, we studied the roles of TRPM8 and TRPA1 in orosensory responding to menthol by subjecting mice gene deficient for either channel to brief-access exposure tests, which measure immediate licking responses to fluid stimuli to capture sensory/tongue control of behavior. Stimuli included aqueous concentration series of (−)-menthol [0 (water), 0.3, 0.5, 0.7, 1.0, 1.5, and 2.3 mM] and the aversive bitter taste stimulus quinine-HCl (0, 0.01, 0.03, 0.1, 0.3, 1, and 3 mM). Concentration-response data were generated from daily brief-access tests conducted in lickometers, which recorded the number of licks water-restricted mice emitted to a randomly selected stimulus concentration over a block of several 10-s stimulus presentations. Wild-type mice showed aversive orosensory responses to menthol above 0.7 mM. Oral aversion to menthol was reduced in mice deficient for TRPA1 but not TRPM8. Oral aversion to quinine was similar between TRPA1 mutant and control mice but stronger than avoidance of menthol. This implied menthol avoidance under the present conditions represented a moderate form of oral aversion. These data reveal TRPA1 contributes to the oral sensory valence of menthol and have implications for how input from TRPA1 and TRPM8 shapes somatosensory-guided behaviors.

## Visual Abstract

**Figure:**
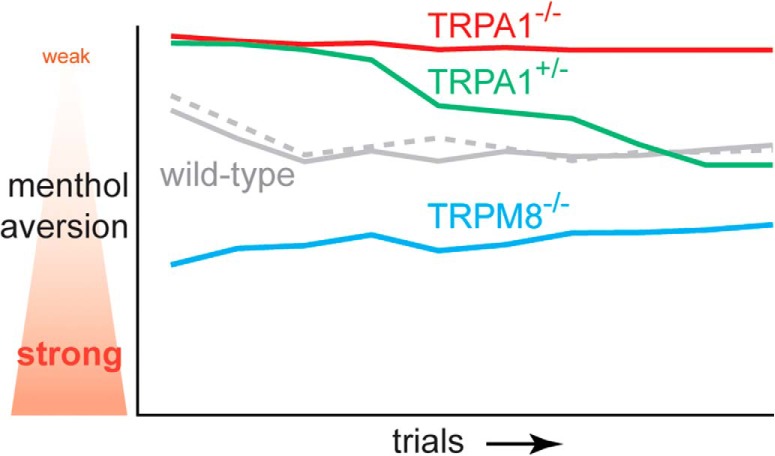

## Significance Statement

Menthol is used in confectionery, tobacco, and oral consumer products to add a pleasant “coolness” to their flavor appeal. However, menthol’s sensation is complex and includes coolness at low but irritation at high concentrations. Elucidating mechanisms that underlie menthol’s aversive flavor component would facilitate understanding of how trigeminal circuits distinguish noxious from innocuous stimuli. Although engaging the cold receptor transient receptor potential melastatin 8 (TRPM8), menthol was discussed to induce oral irritation through its activation of TRP ankyrin 1 (TRPA1), which is expressed on nociceptive fibers usually devoid of TRPM8. Here, we found mice gene deficient for TRPA1, but not TRPM8, show reduced aversion to menthol in an oral sensory-guided behavioral task. These results have implications for how TRPM8 and TRPA1 afferents contribute to hedonic tone during somatosensory-influenced behaviors.

## Introduction

The trigeminal system supplies sensory innervation to cutaneous tissues of the craniofacial region. Peripheral trigeminal sensory neurons can express transient receptor potential (TRP) ion channels implicated for the transduction of noxious stimuli, including the capsaicin receptor TRP vanilloid 1 (TRPV1), which operates as a polymodal nocisensor (Caterina et al., 1997; Tominaga et al., 1998), and TRP ankyrin 1 (TRPA1), which is sensitive to exogenous irritants, such as allyl isothiocyanate (AITC; mustard oil) and allicin (garlic pungency), and implicated to enhance nociceptive transmission (Jordt et al., 2004; Bautista et al., 2005, 2006). Fibers of multiple branches of the trigeminal nerve, including those innervating tissues of high sensory acuity on the cornea and tongue, respond to numerous thermal, mechanical, and chemical stimuli associated with nociception (Carstens et al., 1998). Thus, trigeminal circuits serve a critical role in protective signaling against conditions associated with distress to organs of the head, face, and mouth.

Although frequently considered for their role in nociceptive coding, trigeminal neurons are engaged in part by stimuli that have both aversive and innocuous properties. A notable example of this stems from studies on the oral sensory features of the cooling mimetic and chemesthetic agent menthol, which activates and potentiates lingual nerve fibers sensitive to cooling (Hensel and Zotterman, 1951). Whereas humans report irritation to the oral presence of elevated concentrations of menthol, low millimolar concentrations predominantly elicit only an oral cooling sensation (Cliff and Green, 1994). This result associates with electrophysiology data from rats, where lingual delivery of a low millimolar intensity of menthol capable of activating the lingual nerve (Lundy and Contreras, 1995) was an ineffective stimulus for spinal trigeminal nucleus neurons associated with oral nociceptive processing, although these same cells fired to menthol tested at many fold higher concentrations (Zanotto et al., 2007). Furthermore, long-term fluid intake studies in mice revealed low millimolar intensities of menthol suppressed drinking avoidance of irritants such as nicotine, albeit higher concentrations of menthol were themselves avoided in ingestive tests (Fan et al., 2016).

Delineating mechanisms that support aversion to menthol at elevated concentrations would contribute to unraveling how noxious and innocuous inputs are distinguished by activity in trigeminal circuits. Along this line, the complex sensory features of menthol are associated with its ability to engage multiple receptor mechanisms. Molecular cloning identified that menthol activates the excitatory ion channel TRP melastatin 8 (TRPM8) responsive to cool and cold temperatures, revealing the sensory effects of menthol are partly tied to its actions on a thermoreceptor found on dorsal root and trigeminal fibers (McKemy et al., 2002). Subsequent studies revealed menthol is not selective for TRPM8 but also engages TRPA1, as micro/millimolar concentrations of menthol modulate TRPA1 currents in an expression system and stimulate cultured trigeminal neurons sensitive to the TRPA1 agonist mustard oil (Karashima et al., 2007; Xiao et al., 2008). TRPA1 is typically found on fibers expressing molecular markers associated with nociceptive signaling, such as TRPV1, and only rarely arises in primary sensory neurons that coexpress TRPM8 (Story et al., 2003; Jordt et al., 2004; Kobayashi et al., 2005; Nguyen et al., 2017). It has been proposed that the irritant sensory features of menthol are linked to TRPA1 activation (Karashima et al., 2007; Fan et al., 2016). Accordingly, trigeminal nerve-mediated respiratory irritation to menthol vapor, as reflected by acute depressions in breathing rate, is nearly absent in mice gene deficient for TRPA1 (Willis et al., 2011). However, involvement of TRPA1 with oral sensory aversion to menthol has not been studied. Further, TRPM8 is evidenced to arise on subsets of primary somatosensory neurons that activate to cooling and noxious mechanical stimuli (Jankowski et al., 2017) or express the nocisensor TRPV1 (McKemy et al., 2002; Dhaka et al., 2008; Nguyen et al., 2017), which leaves the possibility that menthol activation of TRPM8 engages neurons mediating aversive coding.

To investigate the roles of TRPM8 and TRPA1 in sensory-guided behaviors to menthol, we subjected mice genetically deficient for either ion channel and controls to brief-access stimulus exposure tests to measure licking avoidance behaviors to water-soluble concentrations of menthol. Brief-access tests captured immediate licking responses to limited volumes of stimulus fluid to focus on orosensory guidance of ingestive behavior (Davis, 1973; Smith, 2001; Boughter et al., 2002; Ellingson et al., 2009). For comparison, tests were also conducted using the bitter tastant quinine, which causes strong concentration-dependent aversion in brief-access assays. The present work extends prior mouse long-term ingestive studies with menthol and has implications for how the balance between TRPM8 and TRPA1 afferents contributes to somatosensory-guided behaviors.

## Materials and Methods

### Mice

These studies used a total of 99 mice, the majority of which were between two and five months old, originating from The Jackson Laboratory (JAX). Initial body weights and other details of the mice, including line abbreviations, are given in Table 1. Lines included mice with homozygous genetic deficiency for *Trpm8* (TRPM8^KO^) or *Trpa1* (TRPA1^KO^), mice heterozygous for *Trpa1* (TRPA1^HET^), and two approximate control strains (B6 and B6129; JAX Mice Database). Homozygous null TRPM8^KO^ mice show a selective loss of functional TRPM8 expression in neural tissue, including trigeminal ganglion neurons, and display deficits in behavioral detection of cold stimuli and neural sensitivity to menthol (Bautista et al., 2007). The TRPA1 mutant mice lack the pore domain of TRPA1, with expression of functional TRPA1 absent in homozygous null TRPA1^KO^ mice and reduced to 50% in heterozygous TRPA1^HET^ mice (Kwan et al., 2006). Normal drinking avoidance of water flavored with the TRPA1 agonist mustard oil is disrupted in TRPA1^KO^ mice but only partly reduced in TRPA1^HET^ mice, suggesting that the residual TRPA1 expression in the heterozygote line supports an intermediate phenotype (Kwan et al., 2006).

**Table 1.** Mice used in studies with menthol and quinine herein

Line	JAX stock number	Genetics	Abbreviation	*n*	Initial weight, grams (mean ± SD)
C57BL/6J	000664	Approximate control	B6	32	26.5 ± 3.7
B6129PF2/J	100903	Approximate control	B6129	18	32.1 ± 3.1
B6.129P2-*Trpm8^tm1Jul^*/J	008198	Homozygous deficient for TRPM8	TRPM8^KO^	10	23.6 ± 4.6
B6;129P-*Trpa1^tm1Kykw^*/J	006401	Homozygous deficient for TRPA1	TRPA1^KO^	21	26.2 ± 4.3
B6;129P-*Trpa1^tm1Kykw^*/J	006401	Heterozygous for TRPA1	TRPA1^HET^	18	32 ± 3.9

With the exception of 10 B6 mice bred locally, all animals used in these studies were obtained directly from JAX. Mice were experimentally naive at study outset and participated in data collection for either menthol or quinine. An equal number of males and females was tested in the experiment below that compared brief-access licking responses to concentration steps of menthol between TRPM8^KO^ and B6 mice. Due in part to supply constraints, all mice used in other experiments were males.

Mice were housed in a quiet, windowless colony room within a controlled-access, veterinarian-supervised animal facility. The colony room maintained a 12/12 h light/dark cycle and, on average, an ambient temperature of 20°C with 47% humidity. Before experimentation, mice of the same line and sex were group housed in polypropylene “shoebox” cages and provided *ad libitum* access to rodent chow and tap water through, respectively, a conventional wire bar cage lid and overhead bottle with sipper tube. All procedures involving mice were approved by the university Institutional Animal Care and Use Committee and performed in accordance with National Institutes of Health guidelines.

### Apparatus

Six lickometer devices (MS-160, DiLog Instruments) were used to train mice to lick a sipper tube for fluid access and then measure their licking responses to a chemical stimulus delivered across a series of brief-access trials. On a given trial, each lickometer made available to a mouse one of multiple sipper tubes filled with solutions via a small port that opened to a metal/Plexiglas chamber housing the animal. The mouse chamber of each lickometer was modified for these studies by adding a Plexiglas barrier that restricted the free movement of animals to an approximate 10 × 15-cm area facing the fluid access port. A computer running proprietary software that communicated with the lickometer recorded tongue contacts with the metal tip of the sipper tube during licking and the time intervals (precision = 1 ms) between consecutive licks (interlick intervals). The amount of time that the sipper tube was available through the access port could be programmed and was controlled by the opening of a normally closed, computer-actuated shutter. This feature facilitated presentation of solutions to a mouse for only a few seconds to support measurements of brief-access licking behavior. By indexing initial licking responses to stimuli over short periods, brief-access procedures intend to focus on oral sensory influences on ingestive behavior and to mitigate post-oral feedback (Davis, 1973; Smith, 2001). Lickometer devices were located in a dedicated suite of quiet, windowless, small rooms within the controlled-access animal facility that were situated down the hall from the colony room. Squads of four to six mice (one per lickometer) were ran in these studies at one time. Squads always included equal numbers of mutant and wild-type mice to control for temporal factors.

### Brief access procedure

During experiments, individual mice were single-housed in shoebox cages within the colony and placed on a water restriction schedule to motivate behavioral responding, as below. Food was always freely available to mice while in their shoebox cages. Body weights were measured daily. For data collection, mice were transported in their cages between the colony room and the lickometer suite; mice were never removed from the controlled-access animal facility. Daily experiments were started in the morning, in most cases near the beginning of the colony light cycle/end of dark phase.

#### Training

Mice were restricted from drinking water for at least 18 h before the onset of training. During training, experimentally naive mice learned to receive fluid in the lickometer over a 4-d period. On the first and second day of training, mice were given 30 min free access to a single sipper tube of water to familiarize them with accessing fluid in the lickometer device. Each 30-min period began when the mouse made its first lick on the sipper tube; mice were allowed 30 min to make their first lick. On the third and fourth day of training, mice were presented with 20 10-s presentations (i.e., trials) of water to familiarize them with receiving and consuming fluid under a brief-access procedure in the lickometer. On each trial, the shutter that blocked access to the sipper tube was opened and mice were given 30 s to make their first lick, on which the 10-s fluid access period began, and lick data were recorded by the lickometer computer. A response of zero licks was recorded and the trial terminated if a mouse failed to make one lick within 30 s of shutter opening. At the end of the trial, the shutter gently closed to block access to the sipper tube and the lickometer advanced to the next trial in the tube presentation sequence with an intertrial interval of 10 s. Each mouse was run in the same lickometer across all training and test days.

The intent on training days was to have mice consume all of their daily water in the lickometer devices. However, additional free access to water, for ∼1 h via overhead bottle, was given to individual mice following daily training if either their body weight was below 80% of their baseline body weight recorded before water restriction or they made zero licks. Following completion of all training days, all mice were given free access to water via overhead bottles over a 2-d period before testing commenced.

#### Testing

Mice were restricted from drinking water for at least 18 h before the onset of testing. For menthol studies, individual TRPM8^KO^, TRPA1^KO^, TRPA1^HET^, and wild-type mice were evaluated for brief-access licking responses to an approximate one-sixth log step concentration series of room temperature aqueous solutions of menthol [0 (purified water vehicle), 0.3, 0.5, 0.7, 1.0, 1.5, and 2.3 mM (−)-menthol; Sigma-Aldrich]. Brief-access testing occurred over seven consecutive days, where each daily test block consisted of 20 10-s presentations of a single concentration of menthol selected at random, without replacement, for each mouse. Only one concentration of menthol was tested daily to mitigate potential carryover phenomena across different concentrations tested in one block, as menthol can induce lingering effects on trigeminal neurons (Kosar and Schwartz, 1990; Lundy and Contreras, 1995; Zanotto et al., 2007). The structure of each test trial was the same as outlined above for the brief-access training trials. Each 20-trial test block took ∼20 min to complete. To randomize any potential sipper tube differences and effects, each lickometer shuttled between three bottles of stimulus solution, selecting one at random for presentation to the mouse on a given trial. After each session, the lickometer behavioral response chambers, including lids and underneath catch trays, were thoroughly cleaned with water and allowed to dry. The sipper tubes and associated glass bottles were thoroughly rinsed with purified water and allowed to dry. During all test days, mice were maintained under a partial water restriction schedule, where after at least 1 h after testing they received 1 h of free access to water in their home cage via the overhead water bottle.

Menthol concentrations were selected based in part on prior mouse long-term intake data with menthol (Fan et al., 2016), their potency to engage TRPM8 and TRPA1 (McKemy et al., 2002; Karashima et al., 2007) as discussed below, and solubility in water. The highest-tested concentration, 2.3 mM, approaches the saturation limit for menthol dissolved in only water. Stock solutions of the highest menthol concentration were mixed in sealed and covered volumetric flasks for 7 d to ensure menthol dissolution. Lower concentrations were diluted from stock solutions on the morning of testing.

To confirm phenotype in a brief-access setting, a subset of the TRPA1^KO^ and wild-type mice that completed the menthol studies were subjected to retraining and additional brief-access tests with room temperature aqueous solutions of the TRPA1 agonist mustard oil [0 (purified water vehicle), 0.1, 0.3, 0.5, 0.7, and 1.0 mM AITC; Sigma-Aldrich]. In drinking assays, the selected concentrations of AITC are aversive to wild-type mice in proportion to concentration but are readily consumed by mice genetically deficient for TRPA1 (Everaerts et al., 2011), reflecting the diminished sensitivity to mustard oil of the mutant line. Solutions of AITC were mixed and contained in covered or brown glass volumetric flasks and bottles. Animals were allowed a rest period with free access to food and water before the onset of retraining, which refamiliarized the animals with the lickometer apparatus, and subsequent AITC testing. For consistency, training and testing for AITC sessions were performed as above for menthol, including implementation of the water restriction schedule. A single concentration of AITC was tested daily.

To address the modality specificity of the effects of TRPA1 on behavior and to compare menthol results to data for a stimulus known to induce orosensory avoidance in mice, additional squads of experimentally naive TRPA1^KO^, TRPA1^HET^, and control mice were tested for brief-access licking responses to a room-temperature, approximate half-log step concentration series of the bitter taste stimulus quinine [0 (purified water vehicle), 0.01, 0.03, 0.1, 0.3, 1, and 3 mM quinine-HCl; Sigma-Aldrich]. Brief-access testing with quinine was as described above for menthol, with a single concentration tested daily. Aqueous solutions of quinine were mixed in covered volumetric flasks and stored in brown glass bottles before loading into the lickometer sipper tube bottles. Concentrations were selected based on prior brief-access lickometer studies that reported concentration-dependent avoidance of quinine in B6 mice (Boughter et al., 2005; Ellingson et al., 2009).

### Data analysis

The software for each lickometer saved into a single text file the latency of a mouse to make its first lick on the sipper tube and subsequent interlick intervals during each of the 20 trials of a daily stimulus access session. The number of licks emitted on each trial was calculated by adding 1 to the number of interlick intervals that were >50 ms. This criterion aimed to filter any erroneously recorded phantom/noise “licks” (Ellingson et al., 2009).

For menthol studies, latency to first lick gauged the potential influence of oronasal detection of menthol vapor on licking behaviors. The contribution of airborne and olfactory cues to mouse licking in brief-access assays manifests as a systematic change in latency with stimulus concentration (Boughter et al., 2002; Glendinning et al., 2002). For individual stimuli and mice, latency was quantified as the median latency to first lick across the first 5 trials of the stimulus access session, ignoring non-sampled trials (i.e., those with zero licks). Due to positive skew in latency distributions (Jarque–Bera goodness-of-fit tests for normality, *p* < 0.002), latencies were analyzed across concentrations using Friedman’s ANOVA by ranks, which is a non-parametric test for repeated measures. This non-parametric approach did not make assumptions about data distribution shape or equal variance among differences between conditions (i.e., sphericity), as assumed by parametric alternatives.

For individual mice, cumulative lick functions were constructed across sequential trials of a stimulus access session to assess lick rate performance. Each function was standardized by dividing the cumulative licks on each trial by the total licks the mouse completed on trial 20. Cumulative lick functions were compared using the area under each curve. Areas were estimated by taking the approximated integral (trapezoidal method) of the standardized cumulative lick data and analyzed by ANOVA. Before analysis, integral data within each factor level were subjected to 5% Winsorization to accommodate outliers. Under this correction, values more extreme than the lower or upper 5% of data points were, respectively, reset to the smallest or largest values not removed when computing a 5% trimmed mean.

#### Analysis of lick counts and ratios to chemical stimuli

For individual mice, the number of licks they made, defined as their lick count, to a single concentration of a chemical stimulus or water (i.e., 0 mM) was quantified by the median number of licks emitted over the considered trials of the stimulus test session, ignoring non-sampled trials. The majority of analyses were based on licking behavior during the first quarter of trials (i.e., trials 1–5). The first quarter captured behavior when mice were most active on the sipper tubes under baseline conditions, as shown below by analyses of cumulative lick functions for water. This period was sufficient to detect orosensory avoidance behaviors to chemical stimuli including bitter quinine. Furthermore, focusing analyses on the initial 5 trials of a stimulus session intended to further mitigate potential post-oral effects that arose from menthol consumption and accumulation during testing. Along this line, fecal boli captured in the lickometer catch trays and inspected following completion of all 20 menthol test trials could display an unusual sheen or consistency, suggestive of digestive effects.

To account for potential behavioral and activity level differences between animals, the lick count each mouse made to a single stimulus concentration was standardized by dividing this value by the animal’s lick count to water during brief-access testing, arriving at a lick ratio for the stimulus (Boughter et al., 2002; Glendinning et al., 2002; Ellingson et al., 2009). A lick ratio of 1.0 indicates an equal number of licks were made to the stimulus and water. Lick ratios <1 indicate more licks were made to water, with values far below 1 implying strong lick suppression/aversion to the stimulus.

Lick counts and ratios were analyzed across stimulus concentrations using Friedman’s ANOVA by ranks due to skew and unequal variance of data points across levels. Data for individual stimulus concentrations were also compared between TRP channel mutant and control mice to further explore genotype effects. Lick count and ratio distributions for mutant and control animals frequently violated normality, as based on visual inspection of histograms and the Jarque–Bera test (*p* < 0.05). Therefore, between-line statistical comparisons were conducted using two-independent samples Wilcoxon rank-sum tests, denoted here as Wilcoxon tests.

For all analyses above, *p* levels for multiple follow-up and *post hoc* tests were corrected using Holm’s method, which is a modification of the Bonferroni procedure that affords greater power (Aickin and Gensler, 1996). Plots of latencies, lick count, and lick ratio values emphasized showing all data points collected from all mice. In these cases, a conventional bootstrapped 95% confidence interval (CI*) of the center estimate was computed (percentile method; 1000 resamples) and plotted alongside the distribution of points for each stimulus concentration. To assess potential time of day influences on licking, a correlation coefficient was computed between lick counts to water, which gauged baseline licking behaviors in individual animals, and the start times of the water test sessions, expressed as seconds from 00:00 on the test day. Correlation was assessed using the nonparametric Spearman’s rank correlation coefficient (*r*_s_) as input data were not bivariate normal. Proprietary and custom code in MATLAB (release 2018b update 4, MathWorks) was used to mine and calculate response parameters from the database of lickometer text files composed across mice and training/test days, to perform statistical analyses, and for plotting. Parametric and Friedman’s ANOVAs were performed using SPSS (version 23.0.0.2, IBM). All inferential statistical decisions were based on α = 0.05. Final figure configurations were made using Illustrator (version 23, Adobe).

Although infrequent, unavoidable anomalies were encountered during data collection for these studies. These included three mice that licked to water during brief access training, licked during tests with each menthol concentration, but, for unknown reasons, made no licks during the water test session. In these cases, their responses to water on the second day of brief-access training were used for lick count and ratio calculations. Further, two B6129 mice had missing data for select menthol concentrations due to zero licks and were excluded either entirely or in part from statistical analyses as appropriate; sample sizes for each mouse line and condition are noted in the figure captions for clarification. Because of an error in water restriction, a set of data representing 1 d of menthol testing for a squad of six mice was discarded and the mice were retested to collect these data after their remaining test sessions had ended. During the period separating training from testing, cages for two mice were discovered to have leaky overhead water bottles, albeit this was corrected, and the mice ran as normal. Finally, one mouse died during experimentation from a preexisting condition, as based on postmortem veterinary inspection, and its data were discarded.

## Results

**Figure 1.**
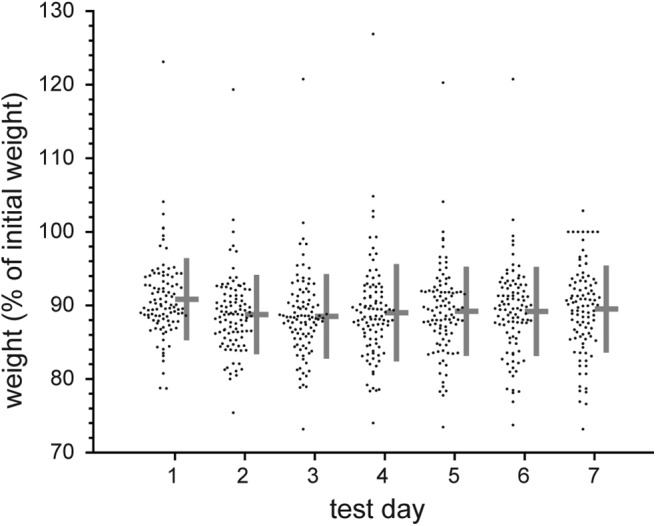
Body weights for individual mice (*n* = 99) on each brief-access test day expressed as a percentage of their initial, pre-water restriction weight (test day weight in grams ÷ initial weight in grams × 100). Data include mice from both the menthol and quinine studies. The cross to the right of each distribution gives its mean (horizontal bar) ± SD (vertical bar), as follows: test day 1, 90.8% ± 5.6%; test day 2, 88.8% ± 5.4%; test day 3, 88.5% ± 5.7%; test day 4, 89% ± 6.6%; test day 5, 89.2% ± 6.1%; test day 6, 89.2% ± 6.1%; test day 7, 89.5% ± 5.9%.

Brief-access ingestive tests to the menthol concentration series were completed on 75 mice in total. An additional set of 24 mice was tested for brief-access responding to the concentration series of quinine. On average, water-restricted mice displayed body weights across test days that, when divided by their initial weight, remained consistently near 90% of baseline value (Fig. 1). Because daily brief-access test sessions did not start at exactly the same time during the morning, we assessed whether licking behavior was associated with time of day. However, across all 99 mice, lick counts to water and the start times of the water test sessions were uncorrelated (*r*_s_ = 0.03, *p* = 0.8), implying time of day did not influence licking.

**Figure 2.**
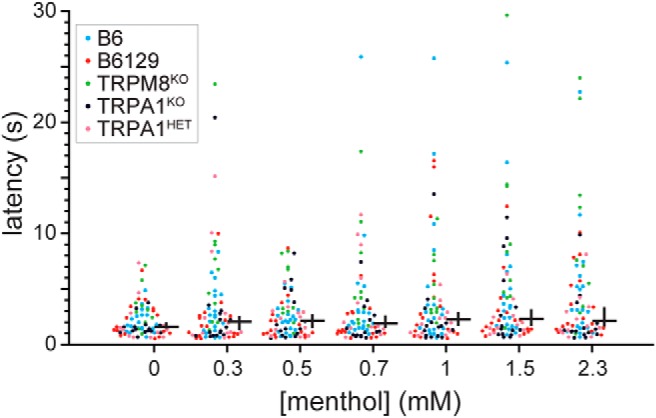
Distributions of latencies to first lick for individual mice of each line across menthol concentrations. The cross to the right of each distribution gives its median (horizontal bar) and 95% CI* (vertical bar). Sample sizes are as follows: 0 mM, *n* = 75; 0.3, 0.5, 1, 1.5, and 2.3 mM, *n* = 74; 0.7 mM, *n* = 73. Holm-adjusted *p* levels for Freidman’s ANOVAs conducted on the influence of menthol concentration on first lick latency for each line follow: B6, 0.45; B6129, 0.16; TRPM8^KO^, 0.19; TRPA1^KO^, 0.77; TRPA1^HET^, 1.

Distributions of latencies to first lick were evidenced to differ across menthol concentrations (Friedman’s ANOVA by ranks, χ^2^ = 20.72, df = 6, *p* = 0.002). However, visual inspection of data points implied latencies only nominally increased from low to high menthol intensities (Fig. 2). Follow-up analyses conducted within each mouse line did not identify a significant influence of menthol concentration on first lick latency (Holm-adjusted Friedman’s ANOVAs, *p* > 0.16). These results implied potential oronasal detection of menthol vapor emanating from the sipper tubes was not a major influence on menthol licking behavior.

**Figure 3.**
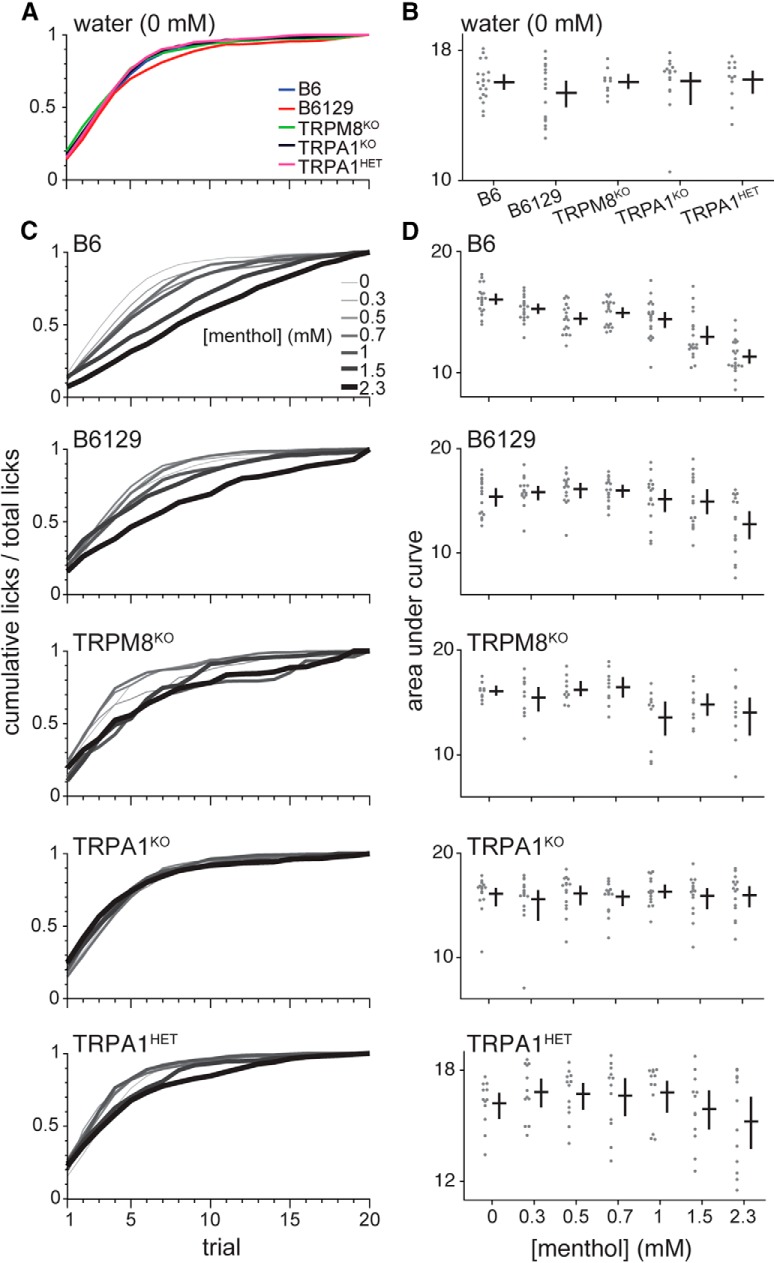
Licking performance of mice in menthol studies. ***A***, Mean standardized cumulative lick functions for water (0 mM menthol) for each mouse line (legend). Each curve tracks mean cumulative licks observed per water trial divided by the mean total licks the line completed at the end of the water access session (i.e., trial 20). ***B***, Distributions of integrals (areas under curves) for water cumulative lick functions that contributed the average plots in panel ***A***. The cross to the right of each distribution gives its mean (horizontal bar) and 95% CI* (vertical bar). One-way ANOVA identified that the mean area under the standardized cumulative lick function to water did not differ across B6 (*n* = 20), B6129 (*n* = 16), TRPM8^KO^ (*n* = 10), TRPA1^KO^ (*n* = 15), and TRPA1^HET^ (*n* = 12) mice (*F*_(4,68)_ = 1.6, *p* = 0.2). ***C***, Mean standardized cumulative lick functions to the menthol concentration series for each mouse line. The curve for each concentration tracks mean cumulative licks per trial divided by the mean total licks mice completed to this concentration on trial 20. The legend in the B6 plot relating line shading/thickness to menthol concentration applies to all panels in ***C***. ***D***, Distributions of areas under the standardized cumulative lick curves for each concentration of the menthol series. Points represent data for the individual mice that composed the average plots for each line in panel ***C***. The cross to the right of each distribution gives its mean (horizontal bar) and 95% CI* (vertical bar). Sample sizes were as stated for panel ***B***. Repeated measures ANOVAs revealed that the mean area under the standardized cumulative lick function to menthol significantly changed with concentration for B6 (*F*_(6,114)_ = 48.3, *p* < 0.001), B6129 (*F*_(2.5,53.2)_ = 9.8, *p* < 0.001), TRPM8^KO^ (*F*_(6,54)_ = 3.6, *p* = 0.004), and TRPA1^HET^ (*F*_(6,66)_ = 3.1, *p* = 0.009) mice. In contrast, the area under the standardized cumulative lick function to menthol did not vary with concentration for TRPA1^KO^ mice (*F*_(6,84)_ = 0.5, *p* = 0.8). For panels ***B***, ***D***, distributions of points show uncorrected data, albeit statistical analyses were performed following 5% Winsorization to accommodate outliers.

Inspection of cumulative licks to water, which was the stimulus that gauged baseline licking behaviors in individual animals, revealed mice of all lines displayed their highest lick rates during the first five of the 20 brief-access trials (Fig. 3*A*). After the 5th trial, the mean rate of licking to water notably slowed and began to plateau. The mean area under the cumulative lick curve for water did not vary across mouse lines (no effect of line on curve integral, one-way ANOVA, *p* = 0.2; Fig. 3*B*). Thus, mice completed the majority of their licks to water within the first quarter of the stimulus access session. Unless mentioned otherwise, analyses of lick counts and ratios that follow were based on responding during this quarter (trials 1–5).

Plots of cumulative licks also revealed that adding menthol to water changed licking behavior in control but not select mutant mice. For instance, B6 mice decreased their initial lick rate to menthol as concentration stepped upward from 0 mM (water). This was evidenced by a generally progressive reduction in the steepness of the early phase of the cumulative lick rate curve for B6 mice (Fig. 3*C*) and a significant reduction in the area under this curve with elevations in menthol concentration (effect of concentration on curve integral, repeated measures ANOVA, *F*_(6,114)_ = 48.3, *p* < 0.001; Fig. 3*D*). These effects implied menthol became increasingly aversive to B6 mice as concentration rose. In contrast, TRPA1^KO^ mice displayed cumulative lick functions to all menthol concentrations and water that steeply approached asymptote by the first quarter of trials (Fig. 3*C*) and did not differ (no effect of concentration on curve integral, repeated measures ANOVA, *p* = 0.8; Fig. 3*D*). The results above began to suggest the oral sensation of menthol became aversive at higher concentrations in a mouse line-dependent manner.

### Wild-type mice show oral aversion to menthol at concentrations above 0.7 mM

**Figure 4.**
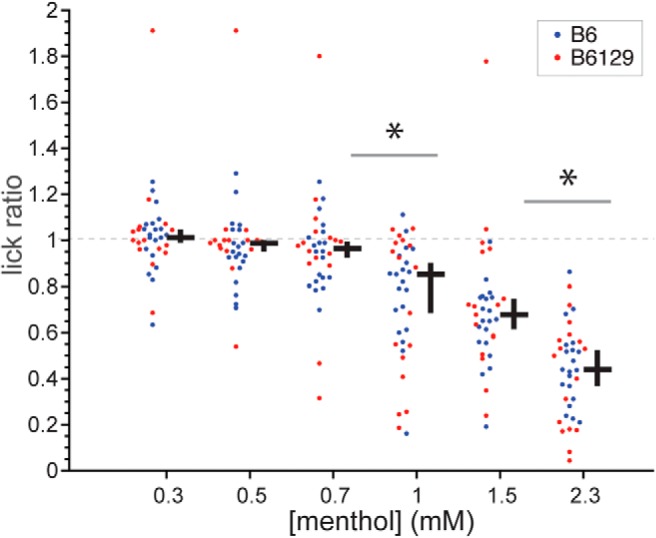
Distributions of menthol:water lick ratios for individual B6 (*n* = 20) and B6129 (*n* = 17 except for 0.7 mM, where *n* = 16) mice across menthol concentrations. The cross to the right of each distribution gives its median (horizontal bar) and 95% CI* (vertical bar). Lick ratios did not differ between B6 and B6129 mice at any menthol concentration (*p* > 0.9). Collapsed across mouse line, lick ratios showed an initial significant decrease when stepping from 0.7 to 1 mM (*p* = 0.02) and further decreased from 1.5 to 2.3 mM (*p* < 0.001), as denoted by asterisks.

Lick ratios to menthol were analyzed for B6 and B6129 mice to explore the implied concentration-dependent avoidance of menthol in control animals. For these mice, lick ratios did not differ between lines at each menthol concentration (Holm-adjusted Wilcoxon tests, *p* > 0.9) but did trend downward in the latter half of the menthol series (Friedman’s ANOVA by ranks, χ^2^ = 107.7, df = 5, *p* < 0.001; Fig. 4). Comparisons of data for adjacent menthol concentrations collapsed across B6 and B6129 mice identified that lick ratios initially decreased in control animals when stepping from 0.7 to 1 mM menthol (Holm-adjusted sign test, *p* = 0.02). An additional reduction in lick ratios was observed from 1.5 to 2.3 mM menthol (Holm-adjusted sign test, *p* < 0.001). Thus, wild-type mice showed significant concentration-dependent avoidance of oral menthol above 0.7 mM.

### Oral sensory avoidance of menthol is contributed by TRPA1 but not TRPM8

**Figure 5.**
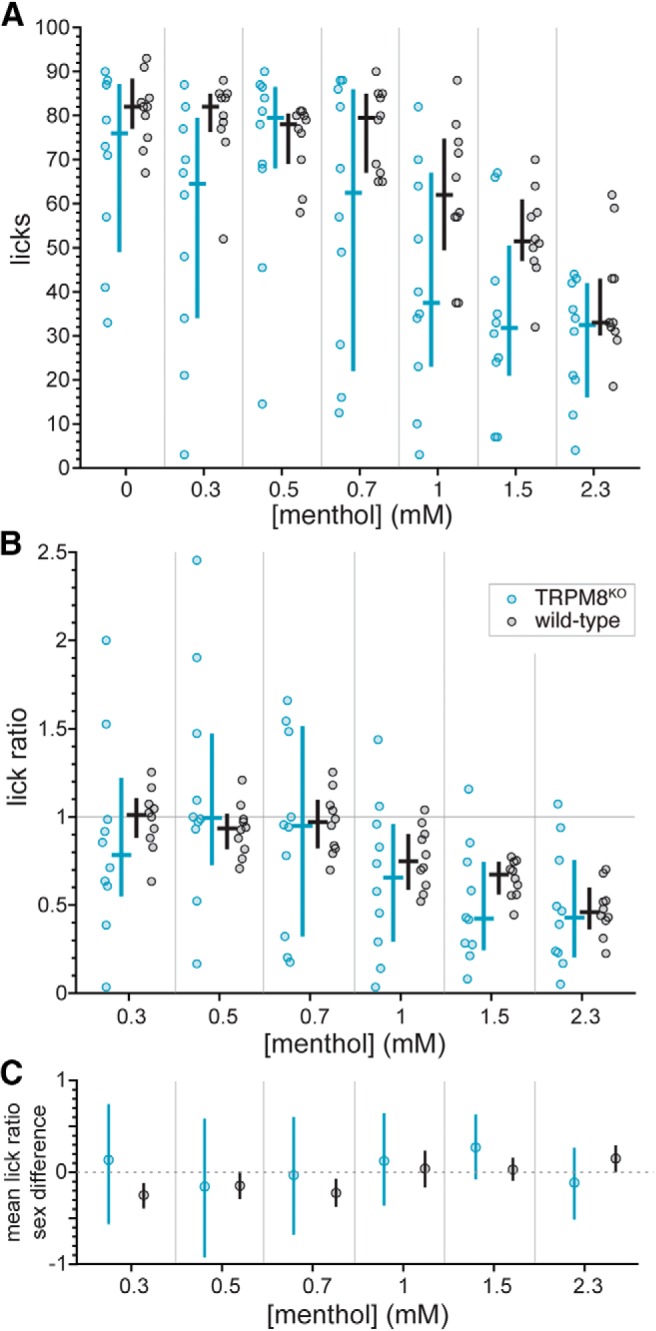
Distributions of lick counts to the menthol concentration series (***A***) and menthol:water lick ratios (***B***) for individual TRPM8^KO^ (*n* = 10) and simultaneously run wild-type (B6 line, *n* = 10) mice. The color-coded (legend, panel ***B***) cross associated with each distribution gives its median (horizontal bar) and 95% CI* (vertical bar). Lick counts and ratios for each menthol concentration did not differ between TRPM8^KO^ and control mice (*p* > 0.16). ***C***, Mean differences in lick ratios between female and male TRPM8^KO^ (five females, five males) and wild-type (five females, five males) mice in panel ***B***. The plotted difference (circle) for each concentration and mouse line (legend for mouse line is given in panel ***B***) was calculated as the mean lick ratio for males minus the mean lick ratio for females. Vertical bar spanning each mean difference represents its 95% CI* based on 10,000 resamples. The trends in this plot suggested that for each mouse line, there was no major influence of sex on lick ratios to the menthol concentration series. Accordingly, statistical analyses collapsed across mouse line to increase analyzed sample sizes revealed lick ratios to each concentration of menthol did not differ between female and male mice (*p* > 0.2).

To gauge the involvement of TRPM8 in concentration-dependent avoidance of menthol, lick counts and lick ratios to the menthol series were compared between simultaneously run wild-type and TRPM8^KO^ mice, with the latter homozygous deficient for *Trpm8*. We reasoned that if input from TRPM8 was responsible for oral aversion to menthol, mice deficient for this ion channel would lack this aversion, as evidenced by no change in licking across menthol concentrations. However, both TRPM8^KO^ and control mice showed reductions in lick counts (Fig. 5*A*) and lick ratios (Fig. 5*B*) to menthol as stimulus concentration increased (Friedman’s ANOVA by ranks: lick counts: χ^2^ > 25, df = 6, *p* < 0.001; lick ratios: χ^2^ > 20, df = 5, *p* < 0.002), with median reductions readily apparent at > 0.7 mM. It is noteworthy that TRPM8^KO^ mice appeared to emit lick counts to select menthol concentrations that were further reduced compared to control (e.g., 1.5 mM, uncorrected Wilcoxon test *p* = 0.03; Fig. 5*A*), implying TRPM8 input normally contributes positively to menthol licking behaviors. However, this observation did not survive α correction for multiple tests, where comparisons of data for each concentration between lines found that TRPM8^KO^ and control mice made the same number of licks (Holm-adjusted Wilcoxon tests, *p* > 0.16) and displayed equivalent lick ratios (Holm-adjusted Wilcoxon tests, *p* > 0.79) across the menthol series. Finally, equal numbers of females and males were included in our squads of TRPM8^KO^ and wild-type mice, although across these animals, lick ratios at each concentration step of the menthol series did not vary by sex (uncorrected Wilcoxon tests, *p* > 0.2; see also Fig. 5*C*). Overall, these results demonstrated that TRPM8 does not contribute to aversive orosensory responses to water-soluble concentrations of menthol.

**Figure 6.**
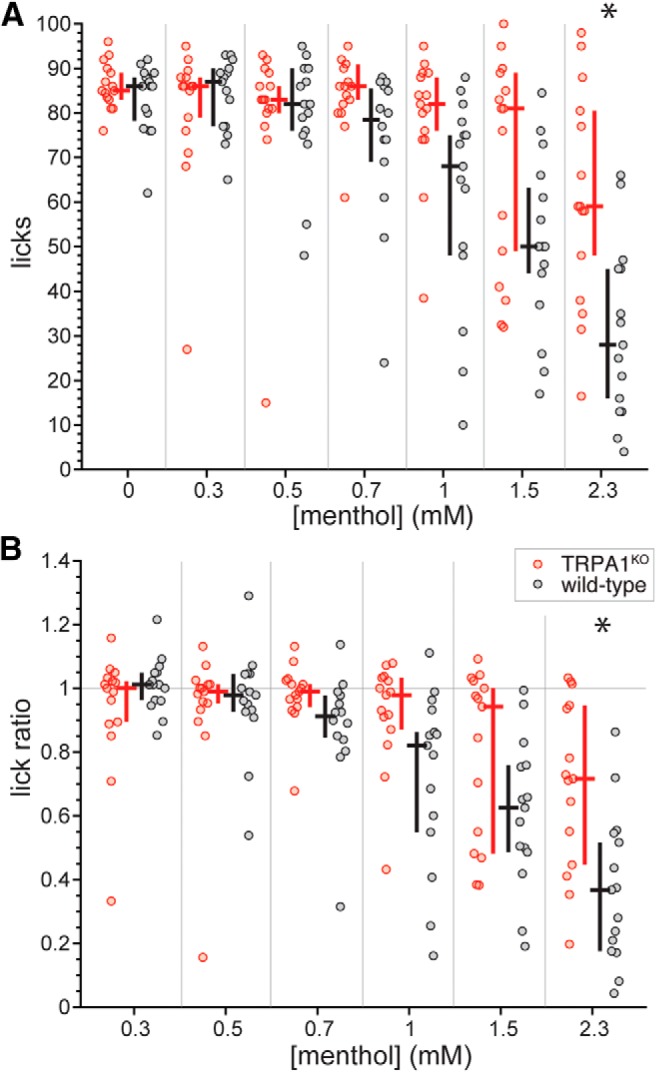
Distributions of lick counts to the menthol concentration series (***A***) and menthol:water lick ratios (***B***) for individual TRPA1^KO^ (*n* = 15) and simultaneously run wild-type (*n* = 16; 10 B6 mice and six B6129 mice) mice. Plots and analyses reflect and accommodated missing data for two B6129 mice: one made no licks from 0.3 to 2.3 mM menthol (i.e., licked only to water) and the other made no licks to 0.7 mM menthol. The color-coded (legend, panel ***B***) cross associated with each distribution gives its median (horizontal bar) and 95% CI* (vertical bar). TRPA1^KO^ mice showed higher lick counts and ratios to 2.3 mM menthol compared to control mice (*p* < 0.03), as denoted by asterisks.

On the other hand, analyses of lick count and ratio data in mice deficient for TRPA1 implicated this ion channel with signaling related to menthol aversion. TRPA1^KO^ mice, which are homozygous deficient for *Trpa1*, showed median lick counts (Fig. 6*A*) and lick ratios (Fig. 6*B*) to menthol that were relatively flat up to 1.5 mM. In fact, the number of licks TRPA1^KO^ mice made to menthol did not significantly vary from 0 (water) to 1.5 mM (Friedman’s ANOVA by ranks, χ^2^ = 6.3, df = 5, *p* = 0.3), with menthol lick ratios also showing invariance up to 1.5 mM (Friedman’s ANOVA by ranks, χ^2^ = 5.4, df = 4, *p* = 0.3) and median values near 1 (indifference from water). These results contrasted with effects observed for simultaneously run wild-type mice, which showed decreasing lick counts (Friedman’s ANOVA by ranks, χ^2^ = 39.3, df = 5, *p* < 0.001; Fig. 6*A*) and lick ratios (Friedman’s ANOVA by ranks, χ^2^ = 29.3, df = 4, *p* < 0.001; Fig. 6*B*) to menthol with increments in concentration up to 1.5 mM, with median reductions in licking becoming notably apparent at > 0.7 mM. Further, whereas TRPA1^KO^ mice appeared to decrease their licks and begin to avoid menthol when the concentration was raised to the highest-tested 2.3 mM, they still made significantly more licks (Holm-adjusted Wilcoxon test, *p* = 0.01) and showed higher lick ratios (Holm-adjusted Wilcoxon test, *p* = 0.02) to 2.3 mM menthol than wild-type controls (Fig. 6). Thus, an absence of TRPA1 causes the orosensory avoidance function for menthol to rightward shift, indicative of reduced sensitivity to menthol.

**Figure 7.**
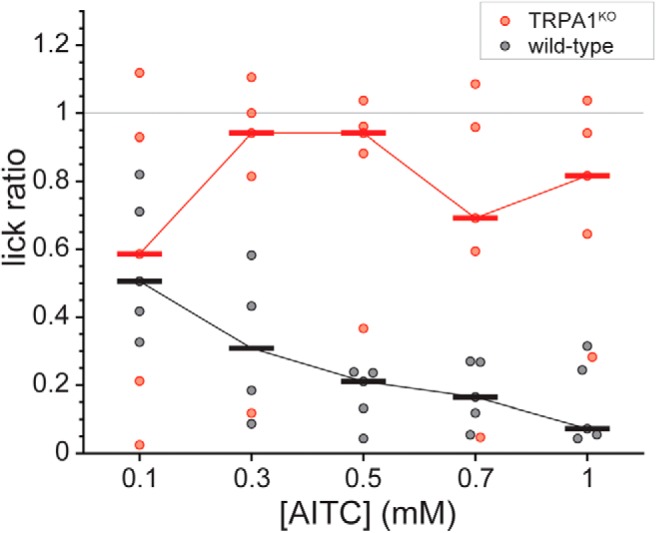
Distributions of AITC:water lick ratios for individual TRPA1^KO^ (*n* = 5) and wild-type (*n* = 5; B6129 line) mice. Plot reflects missing data for one B6129 mouse that did not lick to 0.3 mM AITC. Ratios were calculated using licks to water measured during menthol testing. Color-coded (legend) boldened horizontal bars give median lick ratios for each line across AITC concentrations. Sample sizes were low for statistical analysis, although a trend for concentration-dependent avoidance of AITC was apparent and absent in the median responses of wild-type and TRPA1^KO^ mice, respectively.

To confirm phenotype in a brief-access setting, a subset of the TRPA1^KO^ and wild-type mice that completed menthol testing was subjected to additional brief-access exposure tests with a concentration series of the TRPA1 agonist AITC. Although sample sizes were low for statistical analysis, wild-type mice clearly displayed median lick ratios to AITC that systematically decreased to near zero as concentration was raised to 1 mM (Fig. 7), which is strongly aversive to mice in drinking assays (Everaerts et al., 2011). In marked contrast, this trend was absent in TRPA1^KO^ mice, which generally licked and were indifferent to AITC across concentrations (Fig. 7). The lack of sensitivity to AITC in the mutant group is expected for animals that lack TRPA1 function.

**Figure 8.**
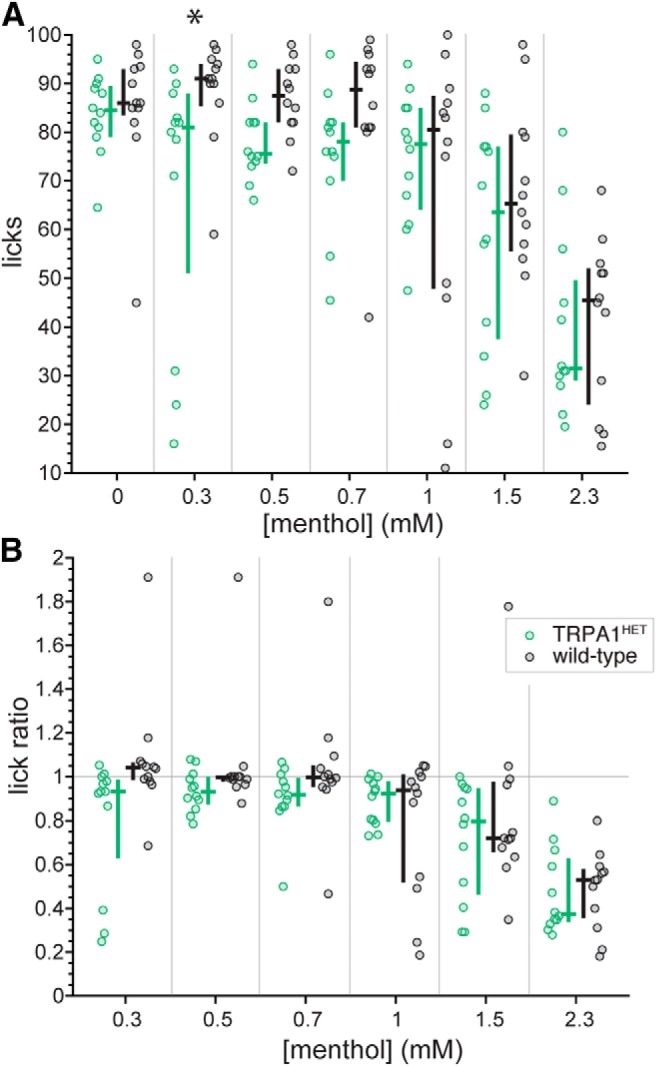
Distributions of lick counts to the menthol concentration series (***A***) and menthol:water lick ratios (***B***) for individual TRPA1^HET^ (*n* = 12) and simultaneously run wild-type (*n* = 12; B6129 line) mice. The color-coded (legend, panel ***B***) cross associated with each distribution gives its median (horizontal bar) and 95% CI* (vertical bar). TRPA1^HET^ mice made fewer licks to 0.3 mM menthol compared to controls (Holm-adjusted Wilcoxon test, *p* = 0.046; denoted by asterisk in panel ***A***), albeit no other line differences were detected.

**Figure 9.**
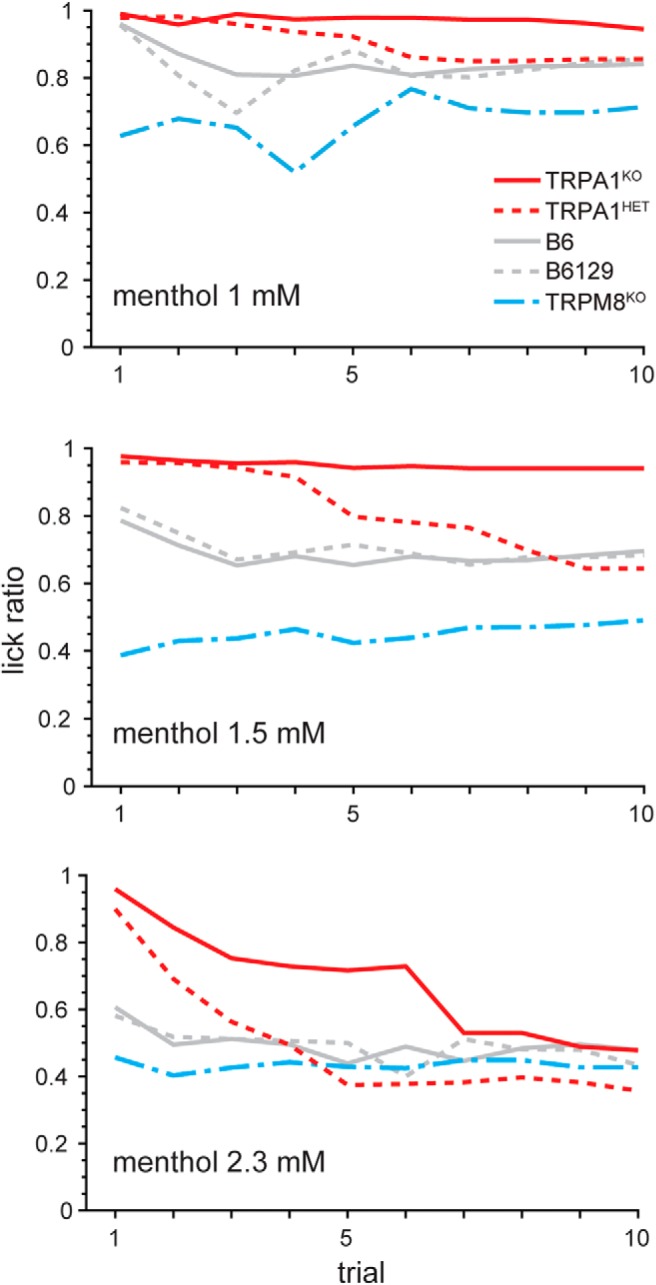
Temporal analysis of menthol licking behavior. Plots track for each mouse line their median lick ratio for the three highest concentrations of menthol as these ratios evolved over the first 10 consecutive trials of test sessions. For each trial of a given menthol concentration, lick ratios for individual mice were calculated as: $\frac{menthol\;lick\;count\;from\;trial\;1\;to\;i}{water\;lick\;count\;from\;trial\;1\;to\;i}$, where i represents the trial number (1–10). Sample sizes were as follows: B6, *n* = 20; B6129, *n* = 17; TRPM8^KO^, *n* = 10; TRPA1^KO^, *n* = 15; TRPA1^HET^, *n* = 12. Although infrequent, a few mice did not respond on the first trial of each menthol concentration, and one B6129 mouse did not respond on the second test trial with these stimuli. These animals were not included in median calculations for trials 1 and 2.

There were no differences between simultaneously run wild-type mice and TRPA1^HET^ mice, heterozygous for *Trpa1*, in lick counts (Fig. 8*A*) or lick ratios (Fig. 8*B*) for 0.7 mM and higher concentrations of menthol (Holm-adjusted Wilcoxon tests, *p* > 0.2). Although this result partly suggested that deficiency for a single *Trpa1* allele does not affect menthol oral avoidance, evidence for an intermediate phenotype became apparent in TRPA1^HET^ mice when licking behavior was inspected with increased temporal resolution. Figure 9 plots for each mouse line how their median lick ratios to the three highest concentrations of menthol evolved over the first 10 trials. The multifactorial time series and non-parametric nature of the data that composed this figure complicated efficient statistical analysis. Nonetheless, median trends visible in this plot implied that TRPA1^HET^ mice uniquely shifted their responding to menthol over trials to arrive at control-like phenotype, with their lick ratios on initial trials showing greater similarity to and de-trending from that of the TRPA1^KO^ line. This pattern was notably apparent for 1.5 mM menthol, where TRPA1^HET^ mice showed median lick ratios similar to those of TRPA1^KO^ mice, near a value of 1 (indifference from water), on early trials but decreased their lick ratios to approximate those for wild-type mice as trials progressed (Fig. 9). Similar trial-dependent intermediate responses by TRPA1^HET^ mice were also apparent for 1 and 2.3 mM menthol (Fig. 9). These observations suggested that heterozygous deficiency for *Trpa1* caused a haploinsufficiency phenotype involved with orosensory avoidance of menthol. Intermediate phenotypes were reported in prior studies of mice heterozygous for *Trpa1* (Kwan et al., 2006, 2009).

**Figure 10.**
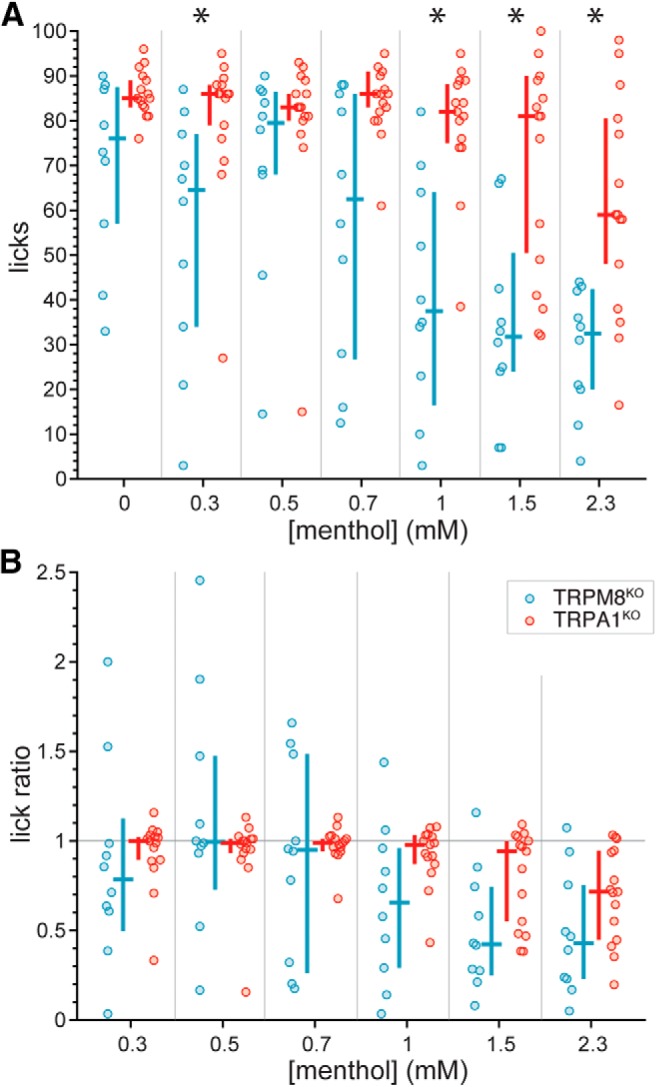
Distributions of lick counts to the menthol concentration series (***A***) and menthol:water lick ratios (***B***) for individual TRPM8^KO^ (*n* = 10) and TRPA1^KO^ (*n* = 15) mice. The color-coded (legend, panel ***B***) cross associated with each distribution gives its median (horizontal bar) and 95% CI* (vertical bar). Data points are re-plotted from Figures 5, 6; note that 95% CI*s do not perfectly match between figures due to re-bootstrapping. As denoted by asterisks in panel ***A***, TRPA1^KO^ mice made more licks to 0.3, 1, 1.5, and 2.3 mM menthol compared to TRPM8^KO^ mice (*p* < 0.03).

Inspection of time-evolved lick ratio data suggested there was a marked difference in menthol avoidance between mice homozygous deficient for TRPA1 or TRPM8 (e.g., 1.5 mM; Fig. 9). Re-plotting data for the mutant lines from Figures 5, 6 revealed TRPA1^KO^ mice showed higher lick counts to 0.3, 1, 1.5, and 2.3 mM menthol compared to TRPM8^KO^ mice (Holm-adjusted Wilcoxon tests, *p* < 0.03; Fig. 10*A*). These line differences in licking, particularly to concentrations >0.7 mM, suggested mice gene deficient for TRPM8 may find menthol less appealing than mice that lack TRPA1 function. Along this line, TRPM8^KO^ animals appeared to show reduced lick ratios to select, high menthol concentrations compared to TRPA1^KO^ mice (e.g., 1.5 mM; Fig. 10*B*; uncorrected Wilcoxon test *p* = 0.04), albeit these observations did not reach significance or persist following α correction for multiple comparisons. Nevertheless, the differences in licking to menthol observed between the mutant lines, and the patterns of oral responding they showed compared with wild-type controls, implied that the TRPA1, but not TRPM8, ion channel contributes to oral aversion to menthol.

### TRPA1 is not involved with aversive oral behaviors to the bitter tastant quinine

**Figure 11.**
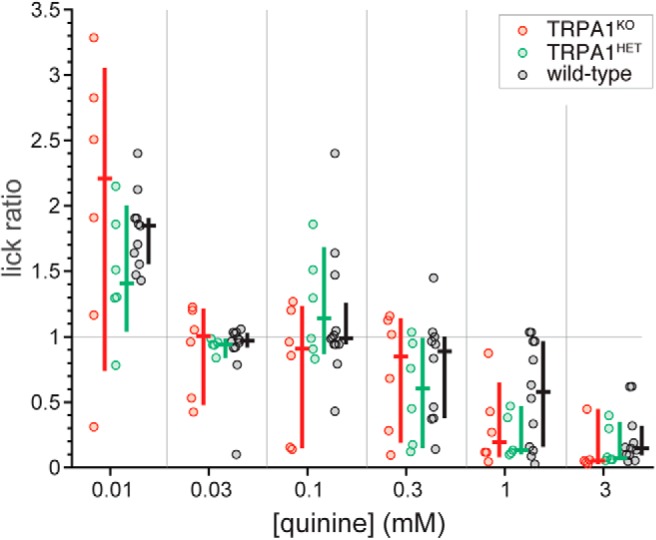
Distributions of quinine:water lick ratios for individual TRPA1^KO^ (*n* = 6), TRPA1^HET^ (*n* = 6), and simultaneously run wild-type (*n* = 12, B6 line) mice. The color-coded (legend) cross to the right of each distribution gives its median (horizontal bar) and 95% CI* (vertical bar). Plots and analyses reflect and accommodated missing data for quinine as follows: TRPA1^KO^ mice, one mouse made no licks to 3 mM; TRPA1^HET^ mice, one mouse made no licks to 1 mM and another made no licks to 0.03 mM; wild-type mice, one mouse made no licks to 0.3 mM, another made no licks to 0.01 and 0.3 mM, and an additional mouse made no licks to 3 mM. No differences were found between lines for lick ratios to quinine (*p* > 0.49).

To address specificity, we evaluated if genetic manipulation of TRPA1 affected orosensory avoidance behaviors to non-chemesthetic stimuli by testing TRPA1^KO^, TRPA1^HET^, and wild-type mice for brief-access intake of a concentration series of the bitter taste stimulus quinine. Across mice, median lick ratios to quinine systematically decreased as concentration rose (Friedman’s ANOVA by ranks, χ^2^ = 62.6, df = 5, *p* < 0.001; Fig. 11), approaching zero at the highest concentration indicative of near complete avoidance. Lick ratios to each concentration of quinine did not differ between simultaneously run wild-type and TRPA1^KO^ or TRPA1^HET^ mice (Holm-adjusted Wilcoxon tests, *p* > 0.49). Further, lick ratios to each quinine concentration did not differ between TRPA1^KO^ and TRPA1^HET^ mice (Holm-adjusted Wilcoxon tests, *p* > 0.89). Thus, although contributing to oral aversion of the chemesthetic stimulus menthol, TRPA1 does not influence oral avoidance of the prototype bitter taste stimulus quinine. These results combined with the orosensory nature of the behavioral assay implied disruption of brief-access avoidance to menthol following knock-out of TRPA1 results from selective impairment of function in normally TRPA1-expressing sensory neurons and not a non-specific effect. These data also provide a reference for comparison of menthol data, discussed below.

## Discussion

Here, we show that sensory-guided aversion toward menthol is contributed by a component feature mediated by the TRPA1 ion channel. Compared to wild-type controls, TRPA1 gene-deficient mice displayed reduced, but not abolished, aversive orosensory responses to aqueous menthol solutions in brief-access exposure tests, which aim to measure licking responses to stimuli in the absence of post ingestive feedback to index sensory/tongue control of behavior (Davis, 1973; Smith, 2001). On the other hand, mice genetically deficient for the cold and menthol receptor, TRPM8, did not show a reduction in orosensory avoidance behaviors to menthol. TRPA1 deficiency had no effect on orosensory avoidance of a non-somatosensory but innately aversive bitter taste stimulus. Altogether, the above findings imply that genetic deficiency of TRPA1, but not TRPM8, disrupts transmission of aversive oral sensory signals for low millimolar menthol in somatosensory circuitry.

Our results align with prior data that mice show concentration-dependent avoidance of aqueous menthol solutions in long-term fluid consumption tests and that genetic deletion of TRPM8 does not lessen this avoidance (Fan et al., 2016). However, there are noteworthy differences to consider between this work and the present studies. Menthol avoidance in the prior long-term assay was documented by intake over 16-h epochs averaged across 4 d (Fan et al., 2016). This extended period would capture ingestive behaviors during post-oral stimulus processing (Davis, 1973; Smith, 2001), with TRPM8 and menthol associated with innervation and modulation of gastrointestinal tract function (Zhang et al., 2004; Harrington et al., 2011; Amato et al., 2013). In contrast, the current analyses that indexed oral avoidance of menthol were primarily based on immediate responding to the stimulus for up to only 50 s (i.e., five trials) to mitigate post-oral effects. This methodological difference may account for discrepancies that emerged between studies. For instance, wild-type mice performing in the long-term exposure test avoided 0.64 mM menthol compared to water and, based on extrapolation, may have avoided even lower concentrations of menthol above 0.32 mM (Fan et al., 2016). On the other hand, wild-type mice ran in the present brief-access tests showed aversive orosensory responses to menthol only at concentrations above 0.7 mM (Fig. 4). Thus, for wild-type mice, their threshold for aversion to menthol may arise at higher concentrations when based on only unconditioned sensory input opposed to additional factors.

Fan et al. (2016) reported that TRPM8 gene-deficient mice consuming fluids in long-term tests shifted their avoidance of menthol to lower concentrations, indicative of increased aversion, and, opposite to wild-type mice, displayed greater avoidance of menthol solutions adulterated with the irritant nicotine compared to nicotine solutions alone. This result was interpreted by the authors to imply that the absence of TRPM8 strengthens, or releases, an aversive property of menthol. Along this line, there was a trend in the present brief-access data for TRPM8 deficient mice to show increased orosensory avoidance to menthol, as median lick counts and ratios to select menthol concentrations appeared lower in TRPM8^KO^ compared to control animals (e.g., 1.5 mM; Figs. 5, 9). However, this trend did not always reach statistical significance or survive α correction for multiple comparisons. Nevertheless, the present results build on the prior long-term ingestive data that TRPM8 signaling does not contribute to avoidance of aqueous menthol solutions, with the current findings derived using an assay focused on sensory-guided behavior.

Although appearing to convey only an innocuous signal for menthol, TRPM8 likely evolved in sensory neurons to detect cooling temperatures, not cooling mimetics. The potency of menthol for mammalian TRPM8 probably reflects its ability, as a natural plant product, to co-opt and stimulate a cooling sensor in animals for evolutionary reasons (Vriens et al., 2008). In light of this, it is important to consider that TRPM8 is documented to have a role in thermal avoidance signaling for extreme cold temperatures. Compared to control, mice deficient in TRPM8 show reduced neural firing and immediate early gene expression in somatosensory ganglia and the spinal cord dorsal horn, respectively, in response to stimulation of skin with temperatures considered to be noxious cold (2° to 0°C, Bautista et al., 2007; Knowlton et al., 2010). Moreover, TRPM8 gene-deficient mice continue to sample cold temperatures as low as 5°C that are strongly avoided by wild-type mice, supporting involvement of TRPM8 with cold nocifensive responses (Knowlton et al., 2010). Accordingly, TRPM8 is evidenced to partly arise on peripheral neurons implicated for nociceptive processing, as a fraction of TRPM8-positve fibers coexpress the capsaicin and noxious heat sensor TRPV1 (McKemy et al., 2002; Dhaka et al., 2008; Nguyen et al., 2017) or generate spikes in response to high-threshold (noxious) mechanical stimuli (Jankowski et al., 2017).

Rather than a response to an extreme stimulus, it is conceivable that avoidance behavior to menthol observed under the present experimental conditions represents a moderate form of sensory aversiveness. This is supported by the incomplete orosensory avoidance of menthol in wild-type mice: whereas their median lick ratio to the highest concentration of menthol was reduced compared to lower concentrations, it was not reduced to near zero indicating absolute avoidance (Fig. 4). In contrast, a reduction in median lick ratio to near zero, which reflects a near absence of stimulus licking, was evident for the highest concentration of the bitter taste stimulus quinine (Fig. 11). Thus, quinine taste appears to induce stronger orosensory aversion than menthol chemesthesis at the concentrations tested. It is curious if greater oral aversion to menthol, and a different result for TRPM8, would arise at concentrations higher than used presently. However, such concentrations would require use of chemical solvents beyond only water and for mice to ingest these solvents, potentially leading to non-specific effects. Nonetheless, the present findings reveal that while orosensory aversion to low millimolar menthol does not involve TRPM8, it does rely in part on signaling mediated by TRPA1, agreeing with proposed role for this channel in menthol irritation (Karashima et al., 2007; Fan et al., 2016).

Although displaying disrupted avoidance of menthol, mice homozygous deficient in TRPA1 showed decreased licks and lick ratios to the highest-tested menthol concentration relative to lower values (Fig. 6). Thus, silencing TRPA1 reduced, but did not abolish, aversion to menthol in brief-access exposure tests. The cause of this residual aversiveness to menthol is not immediately clear, although an obvious question is whether non-somatosensory modalities are involved. There is functional evidence that menthol stimulates dose-dependent responses in olfactory circuits (Takai and Touhara, 2015). However, mice tested under the present conditions did not display a clear trend to increase their latencies to initiate licks as menthol concentration rose (Fig. 2). Such trend may be expected if decisions to lick and aversion were influenced by oronasal detection of menthol vapor, including cues mediated by olfaction (St John and Boughter, 2004; St John and Hallagan, 2005). Further, some electrophysiological data imply oral menthol can engage peripheral processes involved with taste (Hellekant, 1969), albeit there is not consensus across physiologic studies that oral menthol effectively stimulates or produces concentration-dependent activation of gustatory nerves (Kosar and Schwartz, 1990; Lundy and Contreras, 1993). Beyond TRPA1 and TRPM8, menthol is known to activate other molecular effectors associated with somatosensory processing including heat-activated TRPV3 found on the tongue (Macpherson et al., 2006; Nilius et al., 2014), which may contribute to residual behaviors following deletion of TRPA1. A recent study also suggests low-micromolar menthol activates a subpopulation of nociceptors that express the Mas-related G-protein-coupled receptor Mrgprd (Wang et al., 2019), which stimulates curiosity for involvement of Mrgprd signaling in menthol avoidance behaviors. However, other reports show 100 μM menthol is an ineffective stimulus for Mrgprd-positive cells that display characteristics associated with nociceptors (Dussor et al., 2008). Finally, it is important to acknowledge that while brief-access stimulus exposure tests intend to block post-oral effects on licking behavior, they may not perfectly accomplish this, as under this method small volumes of solutions are still ingested by mice. Whether menthol accumulation by mice during testing affected their behavior is unknown, although our analyses primarily targeted only the initial quarter of brief-access trials during stimulus sessions to further mitigate potential post-oral effects.

There was notable variance in lick ratios and aversion to select menthol concentrations across individual mice of the same line, including wild-type B6 or B6129 mice (Fig. 4). Wide variance in lick ratios is not unprecedented and was reported in prior brief-access tests with concentration series of aversive taste stimuli in inbred mice, including the C57BL/6J strain (Glendinning et al., 2002). It is noteworthy that such variance could be reduced in some cases by including data from repeated daily test sessions (Glendinning et al., 2002), which is a common approach used in brief-access exposure studies involving gustatory stimuli (Boughter et al., 2002; St John and Boughter, 2004). In contrast, here only one randomly selected concentration of menthol was proffered daily to mice over the trials of one brief-access test session, with different concentrations tested, without replacement, on other test days. Only a single menthol concentration was tested daily to avoid contiguous presentations of different concentrations of a chemesthetic stimulus known to induce lingering effects on oral trigeminal neurons (Kosar and Schwartz, 1990; Zanotto et al., 2007). While it is conceivable that some of the observed variance in licking behaviors may be reduced if this study were extended to include data from additional test days, such extension may also cause non-specific effects of experience from further exposure to and consumption of menthol. Nevertheless, the prior observation that variance in lick ratios can be reduced through inclusion of data from multiple sessions was not associated with a change in the mean/center values of lick ratio distributions (Glendinning et al., 2002), implying the current median trends in menthol licking behavior would persist with additional testing.

Mice gene deficient for TRPM8 showed notably elevated variance in lick ratios to lower concentrations of menthol compared to other mouse lines (Figs. 5*B*, 10*B*). Our studies that involved TRPM8 knock-out and wild-type mice uniquely tested both female and male animals, although such variance was not attributable to a sex effect, as lick ratios to the menthol series did not significantly differ by sex. It is curious if some of the variance in licking by TRPM8 null mice reflects a unique deficiency in their detection of the thermal component of the stimulus solutions. All fluid stimuli were tested at room temperature approximating 20°C, which is an innocuous cool temperature that, when applied orally, strongly activates a subpopulation of cooling-sensitive neurons in the mouse spinal trigeminal nucleus (Lemon et al., 2016). Mice that lack the cold receptor TRPM8 could be thermo-blind to the mild cooling feature of the fluid stimuli tested here. On the other hand, mice with intact TRPM8 function may sense and, through experience, learn to expect oral cooling when licking room temperature solutions. Such thermal experience and expectation could normally play into reducing licking variability given that repeated exposures to a stimulus can decrease variance in mouse licking behaviors (Glendinning et al., 2002), albeit this remains to be empirically tested. Nonetheless, TRPM8 gene-deficient mice showed a reduced variance for lick ratios, which attempt to accommodate general behavioral differences among animals, to elevated menthol concentrations they avoided that was largely comparable to that of TRPA1 deficient mice (1.5 and 2.3 mM menthol; Fig. 10*B*).

The present results pertain to molecular mechanisms underlying aversive orosensory responses to menthol in mice and, more broadly, relate dual activation of TRPM8 and TRPA1 to somatosensory-guided behaviors given the ability of menthol to engage both of these ion channels. We tested water-soluble concentrations of menthol at micro- to low millimolar levels that affect TRPM8 and TRPA1. At concentrations within this range, menthol can induce inward currents though heterologously expressed TRPM8, held at negative holding potentials, that increase with stimulus concentration, plateauing near 1 mM (McKemy et al., 2002). The actions of menthol on mouse TRPA1 are more complex but include increased current flow during the presence of low micromolar menthol and inhibition (Macpherson et al., 2006) followed by a lingering post-stimulus rebound current at higher intensities (Karashima et al., 2007). That menthol can induce inhibition then, on removal, rebound activation (i.e., an off-response) of mouse TRPA1 was corroborated by multiple methods (Xiao et al., 2008). Further, several studies suggest TRPM8 and TRPA1 are largely expressed on distinct subsets of primary sensory fibers, with TRPA1 arising in neurons that nearly always express markers of nociceptive transmission, such as TRPV1 (Story et al., 2003; Jordt et al., 2004; Kobayashi et al., 2005; Nguyen et al., 2017). Thus, menthol engages heterogeneous subpopulations of somatosensory neurons, with the current results implying that the distribution of activation across them shapes whether the sensory percept of menthol becomes behaviorally aversive. It would be interesting in future studies to determine if inhibitory synaptic interactions between TRPM8 and nociceptive TRPA1 fibers influence oral behaviors to menthol (Fan et al., 2016), similar to the role of such interactions proposed for TRPM8 modulation of pain transmission (Knowlton et al., 2013; Dussor and Cao, 2016).

Finally, results presented here contribute to the developing understanding of the role of somatosensory factors in flavor perception and ingestive behavior, which remains poorly understood compared to progress delineating the biology of the gustatory and olfactory components of flavor. In this pursuit it is important to acknowledge that flavor is a human-described construct that may have limitations for generalizing to other species. Along this line, there are species differences in TRPA1 function to consider, including that rising concentrations of menthol cause sigmoidal excitation of human TRPA1 (Xiao et al., 2008) opposed to the off-response that elevations in menthol intensity induce on mouse TRPA1 (Karashima et al., 2007; Xiao et al., 2008). Although cross-species data can only be cautiously compared, this difference may suggest that TRPA1-mediated neural signals to menthol may build over different time courses in human and mouse trigeminal pathways, which may have implications for how TRPA1 signaling plays into oral hedonic codes. This postulate and the present results warrant further investigations on how sensory neural information generated by TRP ion channels is represented in the brain.

## Synthesis

Reviewing Editor: Julie Bakker, University of Liege Decisions are customarily a result of the Reviewing Editor and the peer reviewers coming together and discussing their recommendations until a consensus is reached. When revisions are invited, a fact-based synthesis statement explaining their decision and outlining what is needed to prepare a revision will be listed below. The following reviewer(s) agreed to reveal their identity: NONE.

Both reviewers have found your paper interesting. I agree with their comments. So please follow closely the comments made by reviewer 1 regarding the significance statement and reviewer 2 about the variability in licking ratios.

Reviewer 1:

This manuscript presents a careful behavioral analysis for the potential roles of two TRPs in orosensory hedonics -- TRPA1 and TRPM8. The authors painstakingly describe the contributions of each of these TRPs to orosensory behaviors (thirst-induced licking) driven by menthol. The results are important, as the authors appropriately discuss, for our understanding whereby our orosensory systems, including trigeminal input, impacts ingestive behaviors.

The paper is well written and edited, figures clear, and the methods astoundingly detailed. The authors also closely interpret their results and address caveats and specificity with additional tests (e.g., the final quinine assay). I'd like to add that what is impressive here in contrast to many of the mutant mouse phenotyping studies in this area is that the authors take a deep dive into really fleshing out the hedonic impacts versus distilling down a complex/dynamic behavior like licking into a few summary graphs. For that as well, this is great work.

I have only two minor suggestions on what is otherwise a solid manuscript.

1)As I understand it, the KO and HET mice originated from JAX and thus methods for genotyping of the mice aren't a necessity for inclusion in the paper. Might the authors simply discuss what we know about the presence of absence of the channels in these lines? I presume the KOs (and possibly HETs) were validated by the generating investigators to truly be depleted entirely of the channels? What is known about the relative proportion of the TRPs studied here which may still be left to remain and transduce stimuli in the nerve fibers of any of these mutant lines? does this impact interpretation any differently than considering them as simply HETs or KOs?

2)while I don't fully appreciate the inclusion of significance statements in J Neurosci and eNeuro, this particular significance statement may benefit from the spirit of the nice sentence in the introduction (lines 65-67) which highlights why it may be insightful at a neurobiological level to unravel mechanisms supporting the bidirectional control of behaviors by menthol depending upon intensity (as the authors nicely do here). As someone not in menthol and orosensory perception research, that statement in the intro spoke to me and may be a clear(er) aspect of significance to others as well.

Reviewer 2:

Menthol is known to have complex and divergent orosensory responses, acting both as a cooling agent and sensory irritant/aversive substance. Menthol activates the cold/menthol receptor, TRPM8, and is well established that menthol's cooling and pleasant response is mediated through TRPM8 activation. At higher concentrations, menthol also activates the sensory irritant receptor, TRPA1, and ion channel in nociceptors, the pain-sensing nerves. It has been widely hypothesized that aversive responsive to menthol, which occur at higher concentrations, are mediated or involves TRPA1. However, the role of TRPA1 in the acute aversive response to menthol, occurring immediately after oral contact, has not been directly demonstrated. Since menthol is widely consumed, and is highly controversial as an additive to tobacco products, knowing the targets of its sensory effects is essential.

Addressing this issue, the authors determine for any direct role of TRPM8 and TRPA1 in menthol's aversive response by examining the contributions of these two TRP channels (TRPM8 and TRPA1) in the orosensory and behavioral responses of mice to menthol. Using global knock-out mice of TRPM8 and TRPA1 to perform orosensory-dependent licking behavior tasks upon brief exposure to menthol, the authors provide convincing evidence for involvement of TRPA1 in the aversive behavior to menthol exposure, and rule TRPM8 in the process. The authors also demonstrate that absence of TRPA1 does not affects responses to other aversive stimuli, such as the bitter chemical, quinine. The authors should also be credited for thoroughly describing the methods and data analysis, for others to easily replicate similar studies.

1. Introduction: Earlier mouse studies (Willis et al., 2011, PMID: 21903934) demonstrated that TRPA1 is essential for mice to produce respiratory irritation responses to menthol. Respiratory irritation responses, as measured by acute depression of respiratory rates in rodents upon exposure, are elicited by trigeminal nerve stimulation, similar to oral irritant response producing aversion behavior. TRPA1-KO mice had a strongly reduced respiratory depression response to menthol exposures in that study. This study should be cited and put in context.

2. The authors in line 327 mention that, with respect to licking behavior (as measured by normalized cumulative licks/session) there are significant differences in the area under the curve with increasing menthol concentration across mouse groups. Area under curves should be plotted for each of the groups and placed next to the standardized cumulative lick function graphs to better represent these significant differences.

3. Changes in lick ratio for WT control mice @1 mM menthol (Figures 4, 5,6,8) are quite variable across experiments (ranging from ~60%-95%), especially since this concentration was demonstrated to being the inflection/transition concentration at which the aversive behavior emanates. This variability was very prominent in figure 8, where the mice did not show aversive response behavior at 1 mM. Please discuss this variability. Was the lick ratio dependent on the time of the day the experiment was conducted or the activity period of the mice? In the methods section, in line 148-149 it is indicated that “Daily experiments were started in the morning, in most cases near the beginning of the colony light cycle/end of dark phase.” Do you have any data to show that beginning of the colony light cycle was optimal to conduct the study?

4. There was a large spread in the #of licks and lick ratios between various TRPM8 knock out mice in figure 5 (and in figure 10 where the data was re-used). Please explain this variability. Though you mentioned that, overall, sex did not play a role, and this was the only gene-mutant mice for which females were also used, can this variability explained for both sexes being included for this data set?

5. Please discuss the residual aversive behavior to menthol observed in TRPA1-/- at the highest concentration tested (line ~531, page 23). What other targets, nerve populations could be involved. For example, MrgprD+ nociceptive neurons were also shown to be responsive to menthol (Wang et al., FASEB J., 2018) .

6. In the discussion, while you describe the complex nature of menthol action on TRPA1 and its relevance to the aversive response to menthol, (line 545-550), please discuss the species differences in TRPA1 response to menthol. It is known that mouse TRPA1 has a bimodal response to menthol, inhibiting mouse TRPA1 above 0.1 mM, whereas human TRPA1 remains maximally active. Can mouse data be extrapolated to human responses ?
